# Challenge of a therapeutic sequence: rare case of heart failure in mitral valvular disease intensified by an extreme mediastinal shift from major diaphragmatic eventration

**DOI:** 10.1093/icvts/ivac181

**Published:** 2022-08-03

**Authors:** Françoise Le Pimpec-Barthes, Charles Al Zreibi, Guillaume Reverdito, Pascal Leprince

**Affiliations:** Department of Thoracic Surgery and Lung Transplantation, Hôpital Européen Georges Pompidou, Assistance Publique-Hôpitaux de Paris, Université Paris Cité, France; Department of Thoracic Surgery and Lung Transplantation, Hôpital Européen Georges Pompidou, Assistance Publique-Hôpitaux de Paris, Université Paris Cité, France; Department of Radiology, Hôpital Européen Georges Pompidou, Assistance Publique—Hôpitaux de, Université Paris Cité, Paris, France; Department of Cardiovascular Surgery, Hôpital Pitié-Salpêtrière, Assistance Publique—Hôpitaux de, Université Paris sorbonne, Paris, France

**Keywords:** Diaphragm eventration, Mediastinal shift, Mitral valve repair, Diaphragm plication, Magnetic resonance imaging

## Abstract

Extreme mediastinal shift due to major diaphragm eventration is complex when mitral-valve repair is required. We report the case of a 59-year-old woman with diaphragmatic eventration who had 2 recent episodes of heart failure due to arrythmia associated with severe mitral-valve regurgitation (regurgitant orifice area 47 mm^2^). Forced expiratory flow-volume in the first second and vital capacity (VC) were at 32% and 33%, respectively,decreasing to 20% and 30% when she was in a supine position. We found it impossible to repair the valve first because of the extreme mediastinal shift and respiratory dysfunction. Therefore, we decided to perform diaphragm plication first followed 3 months later by mitral valve repair. Six months after the cardiac operation, the patient showed significant clinical improvement. Forced expiratory flow-volume in the first second and vital capacity increased to 58% and 55%, respectively. The decision to perform the thoracic operation first, followed by the cardiac operation, was the key to improving the patient’s respiratory function and to medializing the heart to safely support cardiac surgery.

## INTRODUCTION

Severe mediastinal shift due to major left diaphragmatic eventration is complex when mitral-valve repair is required. We report the case of a patient with major congenital diaphragmatic eventration surprisingly never treated, who presented with 2 episodes of cardiac failure due to arrhythmia and severe mitral-valve regurgitation. The challenge was to propose the optimal sequence to perform riskless valvular and diaphragmatic surgical procedures.

## CASE

**Figure 1:**
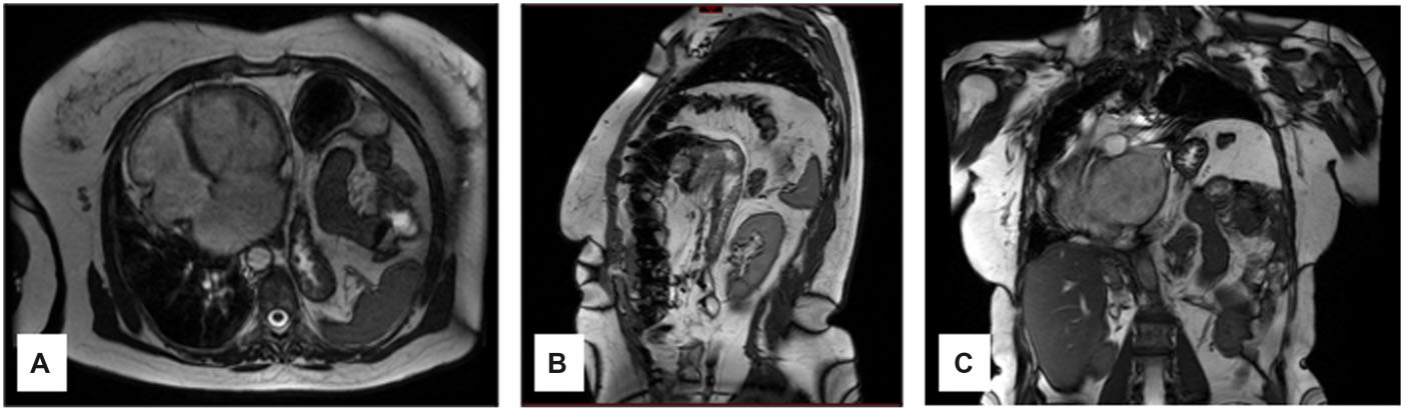
Preoperative dynamic diaphragmatic dynamic MRI (*sequence Fiesta gradient echo steady state GE Architect 3 T 2019)*. (**A**) Axial view showing the mediastinal shift with major left atrial and ventricular dilatation. (**B**) Sagittal view of the major left diaphragmatic eventration. (**C**) Coronal view of a mediastinal shift.

A 59-year-old female with a long history of cardiac arrythmia presented with 2 episodes of cardiac failure that revealed a prolapsed mitral valve causing severe regurgitation (Regurgitant orifice area 47 mm^2^). On echocardiography, the left ventricular function was 52% and the left atrium was dilated to 66 mm in diameter. Mitral-valve surgery was inevitable. However, it was not feasible straight away because of major diaphragm eventration, as seen on dynamic magnetic resonance imaging of the diaphragm (Fig. 1), leading to a complete mediastinal shift. The patient had severe dyspnoea for limited efforts as a result of the combined cardiac and diaphragmatic diseases. Forced expiratory flow-volume in 1 second and vital capacity were measured at 32% and 33% of theoretical values, respectively, decreasing to 20% and 30% in the supine position. We debated the best sequence to perform the 2 procedures safely in this adult patient. One-step surgery (diaphragm and cardiac) including our technique of diaphragm plication added with prosthesis was considered impossible through a median sternotomy. We thought that performing the cardiac surgery first was too risky because of the major mediastinal shift and the significant lung compression. Therefore, we decided to perform diaphragm plication first, keeping in mind the possibility of quickly performing cardiac surgery if necessary. The surgical postoperative course was uneventful apart from left basal pneumonia. Three months later, cardiac surgery was performed including concomitant mitral-valve repair and annuloplasty on both the mitral and the tricuspid valves. Cryoablation of the pulmonary veins was also involved. The postoperative course was marked by reversible cardiogenic shock that required temporary non-invasive ventilation.

Six months after the second surgical procedure, the patient recovered totally. The cardiac and pulmonary results were excellent with normalized cardiac rhythm and respiration (Video 1). The patient is now able to walk for several hours because her breathing has improved. The left diaphragm is in a normal position as seen on dynamic magnetic resonance imaging of the diaphragm (Video 2). The forced expiratory flow-volume in the first second and vital capacity were 58% and 55%, respectively. The patient gave her written informed consent for this report.

## DISCUSSION

In this exceptional case, we report the clinical strategy to cure the mitral valve disease by first reducing the mediastinal shift by diaphragm plication. This sequence was challenging but most likely the only possible strategy. A safe cardiothoracic environment was needed for the first step of diaphragmatic surgery. Similar cases in adult patients were not found in the literature. It is probably the first case of heart failure due to severe mitral valvular disease, complicated by an extreme mediastinal shift due to major left diaphragmatic eventration. The plication rebuilds a strong thoraco-abdominal frontier and removes atelectasis, which increases lung volume even in an almost completely collapsed lung [1, 2]. Therefore, the breathing is improved with excellent long-term results [1, 2]. There are several approaches to perform diaphragm plication but, in our case, the major mediastinal shift contraindicated robotic or thoracoscopic surgery [2, 3]. This first step was uneventful apart from postoperative non-severe pneumonia. Such a complication is common after diaphragm surgery, due to re-expansion of a compressed lung [1, 2]. No cardiac operation was required immediately after diaphragm plication. The patient’s mitral valve could therefore be repaired under optimal conditions. Our therapeutic sequence proposal may be applied in any other combination of diseases involving hemidiaphragm dysfunction. The main goal of this therapeutic sequence was to improve the patient’s respiratory function to support another major operation.

## CONCLUSION

The choice of thoracic and cardiac surgery sequencing was the key to improving the patient’s respiratory function and to medialize the heart to safely support cardiac surgery 3 months later.

Conflict of interest: All the authors declare they have no conflict of interest with the reported subject.
